# Effect of ubiquinol on cardiorespiratory fitness during high-altitude acclimatization and de-acclimatization in healthy adults: the Shigatse CARdiorespiratory fitness study design

**DOI:** 10.3389/fcvm.2023.1129144

**Published:** 2023-07-25

**Authors:** Jie Yang, Xiaowei Ye, Zhen Liu, Mengjia Sun, Shiyong Yu, Hailin Lv, Boji Wu, Chen Zhang, Wenzhu Gu, Jingyu He, Xuhong Wang, Lan Huang

**Affiliations:** ^1^ Institute of Cardiovascular Diseases of PLA, The Second Affiliated Hospital, Army Medical University (Third Military Medical University), Chongqing, China; ^2^ Department of Cardiology, The Second Affiliated Hospital, Army Medical University (Third Military Medical University), Chongqing, China

**Keywords:** cardiorespiratory fitness, study design, cardiopulmonary exercise test, clinical trial, ubiquinol, high altitude

## Abstract

Cardiorespiratory function influences exercise capacity and is an important determinant of high-altitude adaptation. Some studies have investigated the characteristics of changes in cardiorespiratory fitness during high-altitude acclimatization. However, studies on changes in cardiorespiratory fitness during high-altitude de-acclimatization are still lacking and have not yet been elucidated. Furthermore, few drugs have been studied to improve cardiorespiratory function during both processes. The Shigatse CARdiorespiratory Fitness (SCARF) study is a single-center, randomized, double-blind, placebo-control clinical trial to explore the effects of ubiquinol on cardiorespiratory fitness during high-altitude acclimatization and de-acclimatization in healthy adults. Participants will be randomly assigned 1:1 to ubiquinol 200 mg daily or a placebo for 14 days before departure until the end of data collection after return in 7 days. Cardiorespiratory fitness is the primary outcome, while acute mountain sickness and high-altitude de-acclimatization symptoms are secondary endpoints. In addition, laboratory measurements, including routine blood tests and serological measurements, will be performed. To the best of our knowledge, the SCARF study will be the first to reveal the changes in the cardiorespiratory fitness characteristics during high-altitude acclimatization and de-acclimatization. Furthermore, the results of this study will contribute to exploring whether ubiquinol supplementation could be beneficial for endurance exercise capacity at different altitudes and help improve adaptation to acute hypoxia and de-acclimatization.

**Clinical Trial Registration:** This study has been registered in the Chinese Clinical Trial Register (www.chictr.org.cn) as ChiCTR2200059900 and ChiCTR2200066328.

## 1 Introduction

It is known that hypobaric hypoxia is the main characteristic of a high-altitude environment. Acute mountain sickness (AMS) is an environmental illness that can occur in people who arrive at high altitudes above about 2,500 m for the first time. The manifestations of AMS are a series of symptoms involving the brain, heart, gastrointestinal tract, and muscles (1). After returning to a low altitude, reoxygenation may also cause a series of symptoms, named high-altitude de-acclimatization symptoms (HADAS), which are adaptive reactions of the body when facing a normoxic environment (2). Both these symptoms stem from a maladaptation of the cardiovascular and respiratory systems when facing acute hypoxia and reoxygenation. Treatments targeting the cardiovascular and respiratory systems may alleviate these symptoms and help to restore cardiorespiratory fitness back to previous levels.

Reduced arterial oxygenation and increased pulmonary vascular resistance during acclimatization are the main causes of decreasing cardiorespiratory fitness at high altitude (3–6). Arterial oxygenation reduction is attributed to decreased partial pressure inspiratory oxygen and altered coupling of convectional and diffusional oxygen transport systems (7). Meanwhile, the elevated pulmonary vascular resistance is explained through hypoxia-induced pulmonary vasoconstriction and increased cardiac afterload in the right ventricle (8, 9). Although multiple reports have illustrated that the impairment of cardiorespiratory fitness is the main course of decreased physical performance capacity (10–12), there is far less information regarding the characteristics of cardiorespiratory fitness in the process of high-altitude acclimatization and de-acclimatization. Several prior attempts aimed to improve cardiorespiratory fitness after acute high-altitude exposure indicated that selective endothelin A receptor blockade, a PDE-5 inhibitor, and carbonic anhydrase inhibitor are beneficial in reducing cardiac afterload and restoring physical capacity (4, 13, 14). However, the severe side effects of sitaxsentan and acetazolamide limited their clinical applications, especially in elderly and severely ill patients (15, 16). Moreover, few studies have explored the potential medications for the prophylaxis and treatment of high-altitude de-acclimatization-associated symptoms and cardiorespiratory fitness impairment (17). Thus, exploring a new strategy to restore impaired cardiorespiratory fitness and exercise capacity during acclimatization and/or de-acclimatization is necessary.

Coenzyme Q10 (CoQ10) is a fat-soluble antioxidant occurring naturally in the inner membrane of the cell's mitochondria. It plays an essential role as a diffusible electron carrier in the electron transport chain (18, 19). CoQ10 has two forms: the oxidized form and the reduced, active form. This reduced form is widely used in the adjunctive treatment of cardiovascular diseases, nutritional health products, and food additives (20). Notably, CoQ10 plays a key role in myocardial hypoxia, significantly boosting heart health. In patients with myocardial ischemia and hypoxia, ubiquinol, the reduced-form supplement (19, 21, 22), increases aerobic metabolism efficiency and raises the anaerobic threshold. This signifies a lesser need to resort to anaerobic energy production at the same workload (19, 21, 22). Ubiquinol is necessary for the body to produce energy; however, its content in the organism decreases with age and may lead to diseases (23). The lack of ubiquinol causes less efficiency, quick fatigue, and weakened cellular protection ability against oxidative stress (19). These clinical trials implied that ubiquinol could reverse the loss of physical performance ability both at high altitudes and at return to sea level.

We designed this double-blinded, placebo-controlled clinical trial to investigate the hypothesis that ubiquinol supplementation could improve cardiorespiratory fitness, which may be impaired during high-altitude acclimatization and de-acclimatization. This novel Shigatse CARdiorespiratory Fitness (SCARF) study has the potential to identify whether ubiquinol supplementation may rescue the loss of exercise capacity and impaired cardiorespiratory fitness during acute hypoxia and the reoxygenation process. We believe this is the first study that prospectively and accurately evaluates cardiorespiratory fitness during high-altitude acclimatization and de-acclimatization. We also explore the effect of ubiquinol on blood metabolic substrate and automatic nervous function to illustrate the possible beneficial mechanisms further.

## 2. Materials and methods

### 2.1. Setting

This is a single-center, randomized, double-blind, placebo-control clinical trial conducted at Chongqing Xinqiao Hospital and Shigatse Branch Hospital [The Shigatse CARdiorespiratory Fitness (SCARF) Study] in 2022 and registered at www.chictr.org.cn (ChiCTR2200059900 and ChiCTR2200066328). The primary hypothesis of this study is that ubiquinol supplementation may improve cardiorespiratory fitness both at high-altitude acclimatization and de-acclimatization, while AMS and HADAS are secondary endpoints. The study complies with the SPIRIT 2013 recommendations (Standard Protocol Items: Recommendations for International Trials) (24) (Supplementary Material S1). Table 1 depicts the overall schedule and time commitment for trial participants.

**Table 1:** Study schedule and time commitment.

Timepoint	Study period					
	Enrolment	Allocation	Post-allocation			Close-out
	−14 days	0	11 days	17 days	28 days	6 months
Enrolment						
Eligibility screen	X					
Informed consent	X					
Medical and travel history	X					
Vital signs	X					
Allocation/randomization		X				
Interventions						
Ubiquinol group		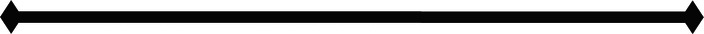				
Placebo group		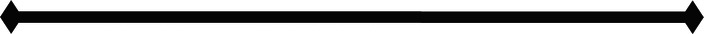				
Assessments						
Baseline variables: age, sex, height, weight			X			
Outcome variables						
CPET			X	X	X	X
Blood routine examination			X	X	X	
Serum marker examination			X	X	X	
The Lake Louise 2018 score assessment				X		
High altitude de-acclimatization syndrome assessment					X	X

### 2.2. Recruitment and study population

Enrolment occurred in Chongqing (low altitude area, 300 m) from June 1st, 2022, and ended on June 15th, 2022. Demographic and medical data of all participants were recorded through interviews. A potential participant must meet all the inclusion criteria and do not meet any exclusion criteria. The inclusion and exclusion criteria are detailed in Table 2. According to previous studies, the risks associated with CoQ10 are minor, even at doses as high as 1,800 mg (25). Moreover, pharmacokinetic studies suggest that exogenous CoQ10 does not influence the biosynthesis of endogenous CoQ10 and is less likely to accumulate in plasma or tissues after the cessation of supplementation (26).

**Table 2:** Inclusion and exclusion criteria.

Inclusion criteria	Age >18 years
	Chinese Han people
	Have no recent (previous 6 months) experience in resistance exercise (including additional cardiopulmonary exercise tests, long-distance running, riding, swimming and skiing and other endurance exercise)
	Are able to perform leg-based resistance exercise
	Are free from musculoskeletal injury (past 6 months)
	Lived at low altitudes (<500 m) for at least 10 years
	Had no recent exposure to high altitude (>2,500 m) (in the last 6 months)
	Willing and capable of providing written informed consent and complying with the study protocol
Exclusion criteria	Incapable of giving informed consent
	Respiratory and cardiovascular diseases
	Liver and kidney dysfunctions
	Malignant tumors
	Coagulation disorders
	A history of AMS
	History of angioedema
	A history of syncope (hypoglycemia excepted)
	Psychiatric disorders or neuroses
	Ubiquinol supplementation with any side-effect in the first 14 days before departure
	Pregnant or nursing women
	A recent history (previous 1-month period) of drug use with acetaminophen, aspirin, nonsteroidal anti-inflammatory drugs
	A recent history (previous 1-month period) of drug use of acetazolamide, diuretics, or other drugs for the prevention or treatment of AMS
	A recent history (previous 1-month period) use of consuming ergogenic aids (e.g., creatine monohydrate), protein-based supplements, or nutritional supplements that may alleviate muscle damage (e.g., antioxidants, polyphenols)
	Other diseases that prevent the patient from going to high altitudes
	Involuntary participant status

The study protocol complying with the Declaration of Helsinki was approved by the Medical Ethics Committee of Xinqiao Hospital of Army Medical University (No. 2022-060-01). All participants had to provide written informed consent after the study details, procedures, benefits, and risks were explained to them verbally and literally. Failure to do so resulted in their exclusion from this study.

### 2.3. Sample size

The G-power tool (version 3.1.9.2, Germany, 2017) was used to calculate the sample size by prior power analysis and to estimate all statistically significant results. The *α* error probability was set at 0.05, and the power (1 − *β*) was set at 0.9. With a correlation among repeated measures of 0.5, a sample size of 39 was determined to be necessary to assess the effects of ubiquinol. A sample size of 36 within two groups (ubiquinol group and placebo group) and within-between interactions were calculated with a nonsphericity correction (*ε*) of 1. Considering a 5% dropout rate, based on a sample size of 39, 41 participants would be required finally.

### 2.4. Intervention

#### 2.4.1. Randomization, blinding, study treatment, and follow-up of participants

Eligible subjects are randomized 1:1 into the ubiquinol group or placebo group by two physicians through a random code generator software (www.randomization.com) to receive either ubiquinol or placebo orally. These two physicians will solely operate randomization and drug distribution, and the randomization information will not be exposed to any participants or researchers, including laboratory staff, data gatherers, and statistical analysts throughout the study. Treatment allocation will be concealed in an opaque envelope opened on the day of treatment. Allocation will not be exposed until the end of the statistical data analysis or when any adverse event, such as severe diarrhea, occurs. A daily reminder email will be set up and sent to participants, and the rest of the medication will be sent back to us to assist in improving compliance. In addition, three serological tests of ubiquinol blood concentration can help monitor whether the participant is consistently taking the medication. The blinding will be maintained throughout the study. Clinical follow-up will be scheduled on days 11, 17, 28, and 6 months after the study began by performing cardiorespiratory tests or filling out self-administered questionnaires for symptoms. The primary outcome is cardiorespiratory fitness, while AMS and HADAS are the secondary endpoints. Safety endpoints include any reported adverse events (Table 3), exercise-limiting symptoms and pre-specified termination criteria (Table 4), and abnormal blood analysis results. The participants will have direct access to receive first aid from their treating team. Any adverse clinical event will be reported to the ethics committee.

**Table 3:** Reported adverse events of ubiquinol (27).

**1. Adverse drug reaction in the digestive system**
Upset stomach
Loss of appetite
Nausea
Diarrhea
**2. Adverse drug reaction in the cardiovascular system**
Palpitation
Hypertension
Hypotension
Tachycardia
**3. Adverse drug reaction in the immune system**
Allergic reactions (rashes, pruritus and hyperhidrosis)
Anaphylactic shock
**4. Adverse drug reaction in the nervous system**
Activation insomnia
Dizziness

**Table 4:** Termination criteria of CPET.

**1. Symptoms or signs of poor breathing and circulation**
Pale face, clammy skin
Cyanosis
Dizziness
Chest pain
Nausea
Severe difficulty breathing
Precardiac pain with ischemic ST changes greater than 2 mm
Severe hypertension (240/140 mmHg)
Systolic blood pressure drop >10 mmHg
**2. Occurrence of severe fatigue or severe leg pain, preventing the participants from pedaling**
**3. Failure of instrument**
More than three electrocardiogram patches fall off
An unexpected power failure
Bluetooth device disconnected
**4. Participant requests for reasons other than above**

### 2.5. Outcomes

The primary outcome will be the cardiorespiratory fitness characteristics both at high-altitude acclimatization and de-acclimatization after ubiquinol supplementation. Specifically, the parameters including peak oxygen consumption (VO_2_), % predicted peak VO_2_, VO_2_ at the anaerobic threshold (AT), peak O_2_ pulse, metabolic equivalents, % maximum predicted heart rate, rate pressure product, VE/VCO_2_ slope, oxygen uptake efficiency slope, VO_2_/work rate slope, cardiac output, exercise ventilatory power, circulatory power, and dead space ventilation / tidal volume at these four visits will be collected for further analysis. A brief definition of these indicators is as follows: peak VO_2_, peak oxygen consumption during an incremental exercise protocol with or without a plateau; % predicted peak VO_2_, peak VO_2_ divided by predicted peak VO_2_; VO_2_ at AT, the value of VO_2_ at AT time; peak O_2_ pulse, the peak oxygen extracted with each beat of the heart; metabolic equivalents, the ratio of exercise metabolic rate to resting metabolic rate calculated as VO_2_/3.5 ml/min/kg; % maximum predicted heart rate, heart rate divided by maximum predicted heart rate; rate pressure product, the product of heart rate and systolic blood pressure; VE/VCO_2_ slope, from exercise onset to peak exercise by the slope of linear regression of VE and VCO_2_; oxygen uptake efficiency slope, slope of the regression line of VO_2_ vs. log_10_ VE; VO_2_/work rate slope, *Δ*VO_2_/*Δ*work rate during incremental exercise; cardiac output, the amount of blood the heart pumps per minute; exercise ventilatory power, the ratio between peak SBP and the VE/VCO_2_ slope; circulatory power, the product of CO and mean arterial BP; dead space ventilation / tidal volume, an estimate of the ventilation-perfusion ratio. In addition, the detailed definitions and calculation methods of these indicators are shown in Table 5.

**Table 5:** The measurement approach for each CPET variable.

CPET fitness measure	Physiological significance	Measurement methodology
Peak oxygen consumption (VO_2_)	“Gold standard” assessment of cardiorespiratory fitness.	The VO_2_ value of plateau attained when there is no further (or relatively small) increases in VO_2_ despite further increases in work rate is determined as VO_2_max (28). Consequently, peak VO_2_ is often used as an estimate for VO_2_max. For practical purposes, VO_2_max and peak VO_2_ are used interchangeably. Symptom limitation during an incremental exercise protocol could be taken to reflect the maximal VO_2_ value even though a plateau in VO_2_ fails to appear (29).
% predicted peak VO_2_	Comparison of peak VO_2_ achieved with predicted value for age, sex, and body size.	Using the Wasserman and Hansen formula (30).
VO_2_ at the anaerobic threshold (AT)	Anaerobic threshold refers to the critical point at which muscle metabolism transitions from aerobic to anaerobic metabolism in an incremental exercise test.	Measurement of VO_2_ and VCO_2_, with AT estimated as the breakpoint in the VCO_2_-VO_2_ relationship using the V-slope method (31).
Peak O_2_ pulse	Reflects stroke volume and peripheral O_2_ extraction. Indicates the peak capacity for oxygen extraction on each heart beat.	Peak VO_2_/peak heart rate.
Metabolic equivalents (METS)	The ratio of exercise metabolic rate to resting metabolic rate. Expresses the relative energy metabolism level of various activities based on energy consumption in quiet and sitting positions (32). 1 Met is defined as the consumption of 3.5 ml of oxygen per kilogram of body weight per minute (33).	VO_2_/3.5 ml/min/kg.
% maximum predicted heart rate	Heart rate response to exercise.	Peak heart rate as a percentage of the predicted peak heart rate calculated using the Tanaka formula (peak heart rate = 208–0.7*age) (34).
Rate pressure product (RPP)	Represents the relative level of myocardial oxygen consumption.	The product of heart rate and systolic blood pressure.
VE/VCO_2_ slope	Determined by the dead space fraction of the breath (VD/VT) and arterial PCO_2_. Typically taken to reflect the degree of match between alveolar ventilation and pulmonary perfusion.	Slope of the regression line of VE vs. VCO_2_ (35).
Oxygen uptake efficiency slope (OUES)	Reflects VO_2_ kinetics.	Slope of the regression line of VO_2_ vs. log_10_ VE (35).
VO_2_/work rate slope	Reflects the metabolic cost of performing external work.	Slope of the regression line of VO_2_ vs. work rate.
Cardiac output (CO)	Measures the strength of cardiac ejection	Using the equilibrium CO_2_ re-breathing technique of Collier (36).
Exercise ventilatory power (EVP)	An integrated index to predict risk representing synergistically linked assessment of ventilatory efficiency and hemodynamics, peak SBP reflecting systemic hemodynamics (37), and VE/VCO_2_ slope reflecting the complex interplay of peripheral and pulmonary abnormalities.	The ratio between peak SBP and the VE/VCO_2_ slope (38).
Circulatory Power (CP)	An index of cardiac systolic function (39).	The product of CO and mean arterial BP (40).
Dead space volume/tidal volume (VD/VT)	An estimate of the ventilation-perfusion ratio representing the efficiency of alveolar ventilation and blood flow matching. The physiological dead space is normally one-third of the tidal volume at rest (36).	[(PaCO_2_ − PECO_2_)/PaCO_2_] − (VDapp/tidal volume) where PaCO_2_ is the arterial CO_2_ pressure, PECO_2_ is the partial pressure of CO_2_ in mixed expired air, and VDapp is the apparatus dead space. PaCO_2_ can be estimated noninvasively using PETCO_2_ (41).

The secondary outcomes will be the questionnaire results of AMS and HADAS. The symptoms of these two diseases are shown in a later section. The effects of altitude and ubiquinol on them will be analyzed in detail in this study. Participants with severe disease symptoms, especially hypoxia-related symptoms, will receive additional treatment.

### 2.6. Study design

**Figure 1:**
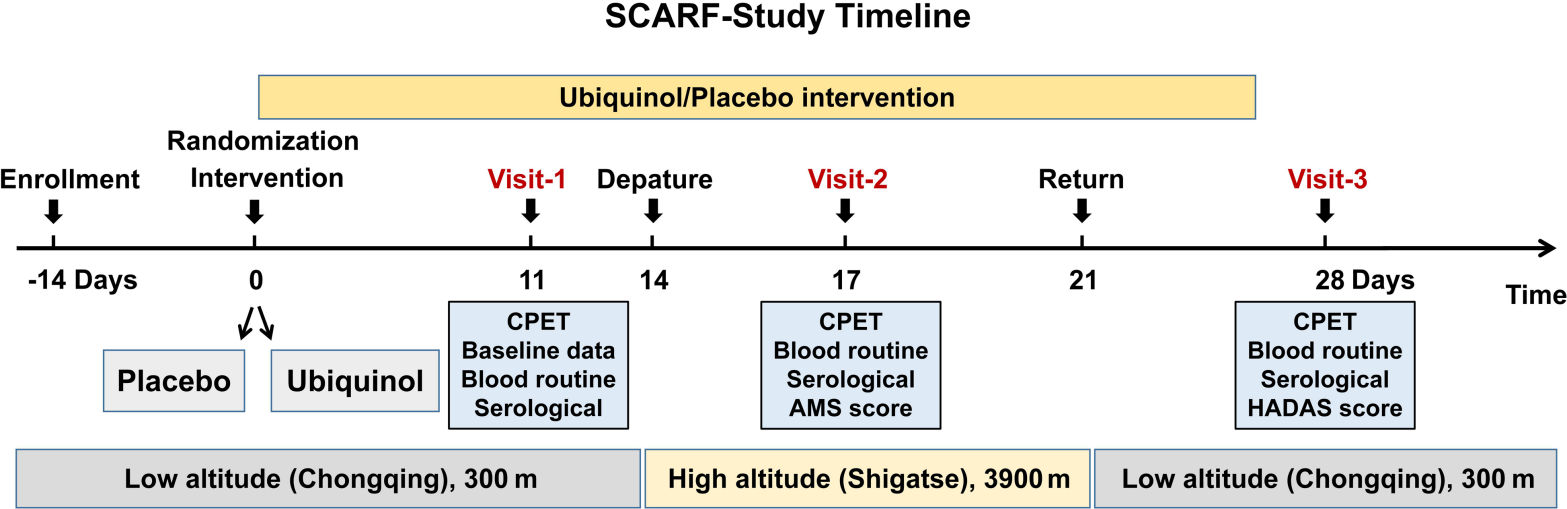
The flow chart of this study.

Figure 1 shows the flow chart of this study. Participants will take ubiquinol orally with a dose of 200 mg daily or a placebo for 14 days before departure. Physical examination, symptoms related to high-altitude acclimatization and de-acclimatization, cardiorespiratory exercise test, laboratory test, and any possible adverse reaction of ubiquinol and exercise will be collected at baseline detection and each visit. During the 14 days of the medication, only participants who did not experience these adverse effects would go to the high altitude as scheduled. It would help us identify related symptoms at high altitudes due to hypoxia rather than adverse events of ubiquinol. The laboratory and exercise tests will be conducted separately on the last three days of the 14 days. Subsequently, they will board a flight to Shigatse (high altitude area, 3,900 m) in 3 h. After arriving at Shigatse, the AMS symptom self-administered questionnaires and adverse reactions of ubiquinol will be followed up daily, and the other prior programs will be carried out on the third day. The subjects will then be flown back to a low altitude after staying for 7 days at a high altitude and scored for HADAS, followed by other prior programs at a low altitude on the 7th day after return. The last visit will be set in the sixth month of this study, which has not yet been conducted. Laboratory tests include a blood routine test of 19 parameters commonly used in the clinic and a serological test of 19 circulating indicators, including glucose, total cholesterol, triglyceride, low-density lipoprotein cholesterol, high-density lipoprotein cholesterol, lactate, non-esterified fatty acid, norepinephrine, epinephrine, plasma renin activity, angiotensin Ⅱ, angiotensin-converting enzyme 2, neuropeptide Y, atrial natriuretic peptide, lactate dehydrogenase, insulin, glucocorticoid, adiponectin, and leptin.

### 2.7. Laboratory tests

Participants will be requested to avoid eating or drinking anything apart from water for up to 12 h. Participants' blood samples will be collected between 8:00 and 8:15 am the day before the exercise tests at different altitudes using the same procedure. After sitting quietly for 15 min, approximately 5 ml of intravenous blood of participants will be collected from the vein at the cubital fossa in the elbow with a tourniquet and mixed immediately with 1 ml of the anticoagulant, dipotassium ethylenediaminetetraacetic acid. Blood samples will be divided into 1 ml and 4 ml parts. The first 1 ml will be analyzed using a BC-3000 plus automated hematology corpuscle analyzer (Shenzhen, China), and the 4 ml will be subjected to high sensitivity enzyme-linked immunosorbent assay kit (Jiangsu Jingmei Biological Technology Co., Ltd., China) for the 19 kinds of circulating parameters. Blood collected at high altitude will be frozen in a −20°C transfer tank and flown to Xinqiao Hospital within 3 h. All blood samples will be measured for biochemical parameters at the Clinical Laboratory of Cardiology Science of Xinqiao Hospital.

### 2.8. The Lake Louise consensus scoring system and high-altitude de-acclimatization syndrome score

The Lake Louise consensus scoring system 2018 version will be used to assess the presence of AMS at high altitudes according to the four main symptoms: headache, gastrointestinal symptoms, fatigue/weakness, and dizziness/vertigo. Scores of 0, 1, 2, and 3 correspond to none, mild, moderate, and severe degrees for each symptom, respectively. AMS is diagnosed as a total score of ≥3, combined with headache (1). We will collect this information every day through self-administered questionnaires at high altitudes.

The HADAS will be evaluated according to the scoring criteria for syndromes, which include complaining of dizziness, fatigue, weakness, headache, blurred vision, lethargy, insomnia, dreaminess, difficulty concentrating, memory loss, slow reaction, and physical signs, including chest tightness, cyanosis, palpitation, slow pulse, precordial pain, changes in blood pressure, decreased appetite, changes in body mass, abdominal distension, diarrhea, edema, constipation, cough, asthma, hair loss, sexual dysfunction, tooth loss, numbness of hands and feet, and skin hemorrhagic spots or ecchymosis (2). In addition, these symptoms do not improve under short-term rehabilitation after organic diseases of the heart, lungs, kidneys, and other organs are ruled out. Physicians who have been systematically trained will score the presence of three or more of the above symptoms or signs according to the following criteria: (1) have a slight reaction but not affect daily work for 0 points; (2) affect work but the symptoms improve soon after rest for score 1; (3) seriously affect daily work and symptoms improve significantly after drug treatment for score 2; (4) seriously affect daily work and symptoms do not improve significantly after drug treatment for score 3.

### 2.9. Respiratory function assessment

Participants will be notified in advance to avoid smoking and consuming caffeinated or heavy meals for at least 2 h before the tests. It will be ensured that the indoor environment is suitable for the entire measurement process [in a dedicated temperature-controlled (20–25°C) laboratory]. Lung function will be assessed using the spiroergometry system with Breath-by-Breath technology (Metalyzer 3B, Cortex, Germany, 2022). Standardized assessments of resting lung function will be performed before respiratory tests during exercises. The best attained values of three attempts of forced expiratory volume in one second (FEV1) and forced vital capacity will be recorded. Before each test, the volume sensor will be calibrated using a standard three-liter syringe, and the gas analyzer will be adjusted with two-point calibration using air and a 16.5% O_2_:CO_2_ mixture. Participants will be provided with a fitting face mask, and volumetric and gas-exchange data will be directly transferred to the metabolic cart software (MetaSoft® Studio, Cortex Biophysik GmbH, 2022), with real-time viewing capacity. Maximum voluntary ventilation (MVV) will be measured from a 12-s maneuver of deep and rapid breathing as the corresponding minute volume. Standards for the performance and interpretation of spirometry data will be obtained from the European Respiratory Society and the American Thoracic Society (42).

### 2.10. Cardiopulmonary exercise test

Before CPET, standardized assessments of resting lung function will be performed. Whole tests will be conducted in a dedicated temperature-controlled (20–25°C) laboratory by a physician and a skilled technician, who is blinded to other procedures and details of this study. The baseline physiological measures for all devices used in this study will be measured for 5 min in a resting state and subsequently in a standing position. CPET will be performed in an erect position through an electronically braked cycle ergometer (EC3000e, Customed, Germany) with Breath-by-Breath technology (Metalyzer 3B, Cortex, Germany, 2022). The cycle ergometry exercise protocol comprises four stages of exercise: 3 min of rest, 3 min of unloaded exercise, a continual increase in resistance of 25 W/min, and recovery with 3 min of unloaded cycling. All patients will continue CPET until the development of limiting symptoms such as angina, dyspnea, or muscular fatigue. CPET will be terminated at the discretion of the operating physician if pre-specified termination criteria develop, such as hemodynamic abnormalities, heart rhythm abnormalities, or neurological impairment.

The AT time is estimated from breath-by-breath data using the V-slope method and validated with other plots using the ventilatory-equivalent method and the respiratory exchange ratio method (31, 43). The parameters for AT will be obtained at this time point. Other peak values will be calculated at identical time points. The calculation method for each indicator detected using the metabolic cart software (MetaSoft® Studio, Cortex Biophysik GmbH, 2022) is described in detail in Table 5.

Standard 12-lead electrocardiogram, blood pressure, and SpO_2_ will be obtained throughout the procedure using a 12-lead connection (Custo-Cardio 3000BT-A, Cortex, Germany, 2022) in real-time, blood pressure cuffs (Suntech Tango M2, Cortex, Germany) in the upper arm and a portable finger clip oximeter (Nonin wristOx2 3150, Nonin, United States), respectively. All these parameters of CPET are the direct output from the cardiopulmonary exercise testing system.

### 2.11. Data collection, management, and analysis

#### 2.11.1. Data collection and management

The data collection tools of this study include oral inquiry, questionnaire, laboratory examination report, and export of cardiopulmonary exercise test report. We have collected relevant information about the participants, including socio-demographic characteristics such as age, sex, alcohol use, tobacco use, medical history, nature and duration of illness, etc. The data, including all primary and secondary outcomes collected at Chongqing Xinqiao Hospital and Shigatse Branch Hospital, will be organized in an Excel spreadsheet in the proper format by a trained research assistant. All the data collected will be stored in Human Plateau Resources Biobank at Chongqing Xinqiao Hospital after being checked by the principal investigator and may only be consulted with the permission of the principal investigator. All personal data will be legitimately stored in encrypted folders and kept unavailable to any third party without the consent of the participants in advance. The data sets generated and/or analyzed during the current study are available from the corresponding author upon reasonable request.

#### 2.11.2. Statistical considerations

Continuous variables will be expressed as means ± standard deviations (SD) or as median (interquartile spacing), and categorical variables as *n* (%) according to the Shapiro–Wilk test. Generalized estimating equations (GEE) with an exchangeable working correlation will be employed. Treatment, altitude, and the treatment multiplied by altitude interaction will be considered independent variables under mixed models. At each altitude, a comparative analysis will be carried out between two groups (ubiquinol group and placebo group), and the least significant difference method will be performed to adjust within-altitude differences for multiple comparisons. Correlation analyses will be performed using Pearson correlation for continuous variables, and Spearman Rank tests will be used between categorical variables. In addition, subgroups will be defined by age for analysis since it is known that ubiquinol levels decrease with age (23). Two-sided tests with *p*-values less than 0.05 will be considered statistically significant. IBM SPSS Statistics software for Windows (version 26, IBM, Armonk, NY, United States) will be used for statistical analyses.

## 3. Discussion

To the best of our knowledge, this study is the first to evaluate cardiorespiratory fitness prospectively and accurately during the high-altitude acclimatization and de-acclimatization process. Moreover, it is a double-blinded, placebo-controlled trial to explore the effects of ubiquinol on cardiorespiratory fitness during acclimatization and de-acclimatization. The clinical trial design depicts cardiorespiratory fitness characteristics at high-altitude acclimatization and de-acclimatization and is designed to reveal cardiovascular and respiratory function restoration during de-acclimatization. In addition, we determine whether ubiquinol supplementation is beneficial for endurance exercise capacity at a high altitude and helps with better adaptation to acute hypoxia exposure. Furthermore, we explore the relationship between the circulating substances and physical performance capacity at high altitudes to be able to account for the possible mechanisms.

The exploration of medicines for high-altitude acclimatization has been underway for decades. A few drugs, such as acetazolamide and *Rhodiola rosea*, have been recommended to promote acclimatization (16, 44, 45). However, limited research focused on the process of de-acclimatization and related symptoms. Thus, in this study, we recruited healthy Han Chinese from Chongqing to assess the possible effect of ubiquinol on cardiorespiratory fitness, AMS, and HADAS symptoms. A previous trial has reported that CoQ10 supplementation with a dose of 90 mg for 6 weeks orally could improve VO_2_max, aerobic, and anaerobic thresholds, indicating the increase of cardiorespiratory fitness of endurance athletes (21). In addition, studies demonstrated that CoQ10 deficiency was positively associated with fatigue, whereas serum CoQ10 levels were inversely correlated with fatigue severity (46, 47). In addition, another meta-regression also confirmed that a daily dose of CoQ10 was associated with fatigue reduction (48). Therefore, we suppose that ubiquinol supplementation at high altitudes may alleviate AMS and HADAS-related symptoms and maintain cardiorespiratory fitness.

Cardiorespiratory fitness reflects the comprehensive ability of the body to transport and utilize oxygen and embodies the overall function of the respiratory and cardiovascular systems (49, 50). VO_2_max is the gold standard for assessing cardiorespiratory fitness and an independent predictor of cardiovascular events and mortality (51–53). Previous studies have indicated that VO_2_max decreases linearly by approximately 8% for every 1,000 m of additional elevation above 700 m, while it descends in an accelerated curve over 6,300 m, suggesting that cardiorespiratory function is impaired at high altitudes (54). However, there is limited information on cardiorespiratory fitness changes during de-acclimatization from a high altitude in healthy individuals, and the underlying mechanisms remain to be elucidated. Thus, in this study, we try to illuminate the possible mechanism by circulating markers of metabolism and the autonomic nervous system (55).

This study has some limitations. First, although this is not a small clinical trial in the CPET trial at a high altitude, this study is limited by a small sample size (*n* = 41) and short follow-up (on days 17 and 28, respectively). Therefore, it is troublesome to implement hierarchical analysis, and these preliminary results need to be confirmed in a large cohort. Second, long-term follow-up may be more meaningful in evaluating ubiquinol supplements on cardiorespiratory fitness.

## 4. Conclusions

This SCARF study will attempt to unravel the mystery of cardiorespiratory fitness during high-altitude acclimatization and de-acclimatization and reveal whether ubiquinol supplementation could be beneficial for physical performance capacity at different altitudes. These preliminary results will provide novel insight into cardiorespiratory fitness and a new pharmacological intervention strategy for a high-altitude exposure population of more than 100 million every year.
