# Supplementary material for: Effect of ubiquinol on cardiorespiratory fitness during high-altitude acclimatization and de-acclimatization in healthy adults: the Shigatse CARdiorespiratory fitness study design
Source: Front Cardiovasc Med. 2023 Jul 25;10:1129144. doi: 10.3389/fcvm.2023.1129144 (PMC10407655; doi:10.3389/fcvm.2023.1129144)
Supplement: Supplementary file 2 [file Table2.pdf]

Ethics approval

<table>
<tr><td>Ethic review number</td><td colspan="4">2022-RD 060-01</td></tr>
<tr><td>Project name</td><td colspan="4">Effect of ubiquinol on cardiorespiratory fitness during high-altitude acclimatization and de-acclimatization in healthy adults: the Shigatse CARdiorespiratory Fitness (SCARF) Study Design</td></tr>
<tr><td>Project source</td><td colspan="4">National Natural Science Foundation of China (Grant No. 81730054)
Chongqing Talents: Exceptional Young Talents Project</td></tr>
<tr><td>Clinical research department</td><td colspan="2">Cardiovascular Department</td><td>Principal investigator</td><td>Huang Lan</td></tr>
<tr><td>Review category</td><td colspan="2">Initial review</td><td>Review method</td><td>Quick review</td></tr>
<tr><td rowspan="2">Review committee member</td><td>Wang Jiang</td><td colspan="3">Deputy Chief Physician and Associate Professor, Department of Cardiology, Second Affiliated Hospital of Army Medical University</td></tr>
<tr><td>Tang Jianlin</td><td colspan="3">Chief Pharmacist, Pharmacological Base, Second Affiliated Hospital of Army Medical University</td></tr>
<tr><td>Submitted documents</td><td colspan="4">Initial Review Application
Research Program (1.0/2022.3.15)
Informed consent (1.0/2022.3.15)
Case Report Form (1.0/2022.3.15)
Statement of economic interests, resumes and copies of GCP certificates of principal investigator
Resumes and copies of GCP certificates of other researchers.</td></tr>
<tr><td>Review decision</td><td colspan="4">Agreed. Frequency of follow-up review: 12 months. Deadline: March 24th, 2023.</td></tr>
<tr><td>Matters need attention</td><td colspan="4">1. Please follow the GCP principle, Helsinki Declaration and the protocol agreed by the ethics committee to develop clinical research and protect the health and rights of the subjects.
2. Before starting the research, please complete the registration or filing of clinical research and other related procedures according to the requirements of relevant functional departments or authorities.
3. In the process of research, if it is necessary to change the main researcher or make any changes to important materials， such as the scheme, informed consent form, recruitment materials and handbooks of researcher, please submit an application for amendment review.
4. If serious adverse events occur in the center, please submit the serious adverse event in time.
5. Please submit the research progress report 1 month before the deadline according frequency of follow-up review; As the leader unit of multi-center research project, the center should also submit the summary report of each center's research and development; Please submit a written report to the ethics committee in time in case of any situation that may significantly affect the trial or increase the risk of the subjects.
6. Please submit a report of violation of the protocol if the included subjects, who do not conform to the inclusion criteria or meets the exclusion criteria, who meet the requirements of stopping the</td></tr>
</table>

	trial but fail to withdraw from the study, who get the wrong treatment or dose, who take in the drugs prohibited by the protocol, fail to follow the protocol to carry out the research, or other situations may have adverse effects on the rights or health of the subjects and violate the scientificity of the research or the GCP principle in the process of research.<br>7. Please submit the suspend report in time to suspend or terminate clinical research in advance.<br>8. Please submit the research completion report if you have completed the clinical research.<br>9. This clinical trial should be implemented within l year from the date of consent. If it fails to be implemented within the time limit, this approval document will be automatically abolished.
**Statement**	The ethics committee reviews the Chinese paper materials submitted for review in strict accordance with China GCP, ICH-GCP and relevant laws and regulations (CD-ROMs and foreign paper materials submitted for review are not reviewed, but only filed), and the review process is not affected by any organization or individual outside the ethics committee.
**Name of ethics committee**	Medical Ethics Committee of Second Affiliated Hospital of Army Medical University, PLA (seal)
**Contact information**	Ethics Committee Office Tel:023-68755422
**Address**	Ethics Committee Office, 3rd Floor, Pharmacy Department, Second Affiliated Hospital of Army Medical University.

**Signature of Vice-chairmen**		**Date**	March 25th, 2023
